# Carbon nanotube‑ and hydroxyapatite‑coated implants: histomorphometric assessment in a rabbit model

**DOI:** 10.1186/s12903-026-08811-8

**Published:** 2026-06-16

**Authors:** Amira S. Eissa, Haitham Mohammed Abou Eleneen, Aya S. Sedik

**Affiliations:** 1 Department of Oral Biology, Faculty of Dentistry, Alexandria University, Champollion Street, Azarita, Alexandria, 21512 Egypt; 2 Department of Oral and maxillofacial surgery, Faculty of Dentistry, Alexandria University, Alexandria, 21512 Egypt

**Keywords:** Osseointegration, Carbon nanotubes, Hydroxyapatite, Titanium implants, Dental coatings

## Abstract

**Objective:**

This study aimed to investigate whether the integration of functionalized carbon nanotubes (CNTs) with hydroxyapatite (HA) could create a bioactive nanocomposite capable of enhancing the early stages of osseointegration. Specifically, we investigated whether the high surface area and nanoscale structure of CNTs, combined with the osteoconductivity of 1% HA, could significantly enhance bone-to-implant contact (BIC) and new bone area (NBA) compared to traditional titanium surfaces in a mandibular model.

**Methods:**

Here we evaluated the osseointegration of titanium implant discs coated with CNTs and CNTs combined with 1% HA in a rabbit mandibular model. Thirty rabbits received uncoated, CNT-coated, or CNT- HA coated titanium discs implanted into mandibular defects. After 4 weeks, bone-to-implant contact (BIC) and new bone area (NBA) were assessed histomorphometrically.

**Results:**

Both CNT and CNT- HA coatings significantly increased BIC and NBA compared to uncoated implants (*p* < 0.05), with no significant difference between coated groups.

**Conclusion:**

CNT-based coatings enhance early osseointegration of titanium implants, while the addition of 1% HA does not confer additional benefit within 4 weeks, suggesting CNT coatings alone may improve implant integration effectively.

## Introduction

Various implant materials have been utilized over the years, including metals, polymers, and ceramics. Early metallic implants, such as those made from gold, silver, and stainless steel, were introduced in the early 1900s but were limited by poor long-term success and short lifespans. The advent of titanium and its alloys marked a significant improvement in dental implantology due to their superior biocompatibility and bioactivity [1]. In recent years, alternative implant materials such as zirconia and alumina have attracted interest because of their favorable esthetic properties and good biocompatibility. Nevertheless, titanium and its alloys remain the most widely used implant materials due to their excellent mechanical properties, corrosion resistance, and well-documented osseointegration [2].

To enhance the biological performance of titanium implants, various surface modification and coating strategies have been investigated to improve osseointegration and bone–implant interaction. Both natural and synthetic coating materials have been explored for this purpose. Natural biomaterials such as collagen and chitosan have been investigated due to their biocompatibility and ability to promote cell adhesion and osteogenic differentiation [3]. In addition, calcium phosphate–based materials, particularly hydroxyapatite, have been widely applied because of their chemical similarity to the mineral component of bone and their osteoconductive properties [4]. On the other hand, several synthetic coatings, including bioactive ceramics, polymers, and nanomaterials, have been developed to improve implant surface characteristics and enhance bone formation [5]. Among these emerging nanomaterials, carbon nanotubes have attracted considerable attention due to their unique physicochemical properties and potential to support bone regeneration [6].

Carbon nanotubes (CNTs), first discovered by Iijima, have since been extensively studied across various fields. Their application in biomedicine has gained momentum due to demonstrated bioactive properties, including promoting bone regeneration [7, 8] and exhibiting antimicrobial effects [9, 10]. CNTs are produced from graphite using methods such as arc discharge, laser ablation, and chemical vapor deposition, resulting in single- or multi-walled tubular structures. They possess unique physical, mechanical, and thermal properties, being extremely lightweight (density of 1.3 g/cm³) while exhibiting high compressive strength (50–63 GPa) [11]. They have attracted significant attention in biomedical field because of their high surface area, exceptional mechanical strength, and ability to support cellular attachment and proliferation [12].

Hydroxyapatite (HA) is one of the most extensively investigated coating materials for dental implants due to its excellent osteoconductive properties and its chemical similarity to the mineral phase of natural bone. HA coatings have been shown to enhance osteoblast adhesion, proliferation, and differentiation, thereby promoting new bone formation and increasing bone–implant contact during the early stages of healing. In addition, HA provides a bioactive surface that facilitates direct bonding between the implant and surrounding bone tissue, which can accelerate osseointegration and improve implant stability [12, 13].

Despite these advantages, conventional HA coatings may present limitations such as coating delamination, mechanical instability, and inconsistent long-term performance. Consequently, recent research has focused on incorporating nanomaterials to enhance the biological and mechanical properties of implant coatings. However, although both CNTs and HA have individually demonstrated promising effects on bone regeneration, limited studies have investigated whether combining CNTs with hydroxyapatite in a nanocomposite coating can further enhance implant osseointegration. Therefore, the present study aimed to evaluate and compare the osseointegration of titanium implants coated with CNTs alone and CNT–hydroxyapatite nanocomposite in an experimental rabbit model.

In this study, the osseointegration of titanium dental implants coated with CNTs and CNTs–HA nanocomposites was evaluated. The null hypothesis tested was that there would be no difference in bone formation and osseointegration among uncoated, CNT-coated, and CNT–HA-coated implants.

## Materials and methods

### Sample size estimation

The minimal sample size was calculated based on a previous study where graphene oxide/ chitosan/ hydroxyapatite (GO/CS/HA) composite coating was fabricated by electrophoretic deposition on Ti substrates. Subsequently, the surface morphology, phase composition, wettability, and bonding strength of this composite coating were researched [14]. The sample size was calculated to detect the difference in bone-to-implant contact (BIC), defined as the primary endpoint of this study among the different studied groups. New bone area (NBA) was defined as the secondary endpoint. Based on Suo et al. [14] results, adopting a power of 80% (β = 0.20) to detect a standardized effect size in the primary endpoint (Bone-to-Implant Contact; BIC) of 0.838, and level of significance 5% (α error accepted = 0.05), the minimum required sample size was found to 10 specimens per group (number of groups = 3) (Total sample size = 30 specimens) [15]. 

Any specimen loss from the study sample due to any reason was replaced to maintain the sample size [16].

### Software

The sample size was calculated using GPower version 3.1.9.2 [17].

### Preparation of titanium specimens

Grade V titanium rods (Bokang Titanium, China), 6 mm in diameter, were cut into discs of 6 mm diameter and 3 mm thickness. The cutting was performed using a CNC wire cutting machine. The diameter and thickness of the discs were then confirmed using a digital caliper (Neiko tools, China).

### Preparation of functionalized carbon nanotubes (CNTs)

Functionalized CNTs were prepared by stirring CNT powder (Shenzhen Nanotech Port, China) with nitric acid at 120 °C for 4 h on a magnetic stirrer and hot plate (F91T, Falc, Italy). The solution was filtered and washed with distilled water, then dried at 55 °C in a muffle furnace (HD-150, Hobersal, Spain) [18].

### Preparation of hydroxyapatite solution (HA)

The wet-chemical precipitation method was performed by adding 0.5 M calcium nitrate tetrahydrate solution to 0.3 M ammonium phosphate in a 1:1 ratio until a white suspension with a Ca/P ratio of 1.67 was formed. Ammonia was added to the suspension until the final pH reached 10, as checked by a pH meter (HQ411D, Hach, USA). The HA suspension was left to age for 24 h, then washed with deionized water and dried [19].

### Preparation of carbon nanotube-hydroxyapatite (CNT-HA) nanocomposites

1g of the functionalized CNTs was stirred with HA in solution form at 60 °C for 5 h to create nanocomposites of CNT-HA with 1 wt% HA. The solution was then dried in a furnace then sintered at 500 °C for 1 h [20].

### Coating of the titanium specimens

The titanium discs were acid-etched using Piranha solution and then sandblasted (Basic eco., Renfert, Germany). The discs were washed with ethanol and cleaned in an ultrasonic cleaner (T-14, L&R manufacturer, USA). Two solutions were prepared: the first containing CNT powder dissolved in 80% ethanol, and the second containing CNT-HA nanocomposite powder in 80% ethanol.

The electrophoretic deposition (EPD) technique was used to coat the titanium discs. A DC power supply (PS-305D, Dazheng, China) was connected with the positive end (anode) to a graphite rod and the negative end (cathode) to a titanium disc. The disc and the rod were inserted in one of the solutions 1 cm apart. The power supply was set at 20 V and 3 A for 15 min. The discs were then removed and air-dried for 24 h. All implants were handled under aseptic conditions throughout preparation and implantation. UVC irradiation was applied immediately prior to surgery as a surface decontamination and photofunctionalization step to reduce microbial load while preserving the physicochemical properties of the CNT and CNT–HA coatings. This approach was selected to minimize potential alterations in coating morphology that may occur with conventional sterilization methods. Previous studies have demonstrated that UVC irradiation can reduce bacterial contamination and enhance the surface bioactivity of titanium implants, although it is primarily considered a decontamination and photofunctionalization method rather than a validated sterilization technique [21].

### Characterization of the coated discs

**Fig. 1:**
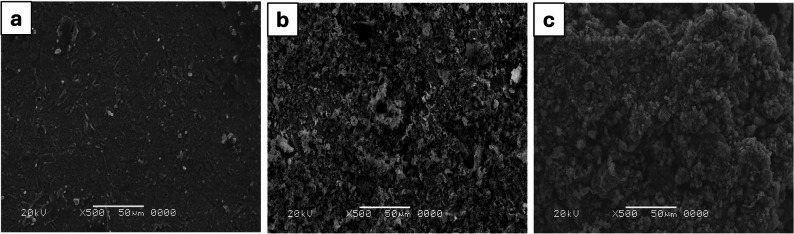
Scanning electron micrographs; **a** show the titanium surface exhibiting roughness and scratches that result from surface treatment with sandblasting and acid etching prior to coating, **b** the titanium surface with flake-like structures of the CNT coat, and **c** the titanium surface coated with the CNT-HA nanocomposite where amorphous structures of CNT are entangled with more regular structures of HA. Scale bar = 50 μm is ×500

**Fig. 2:**
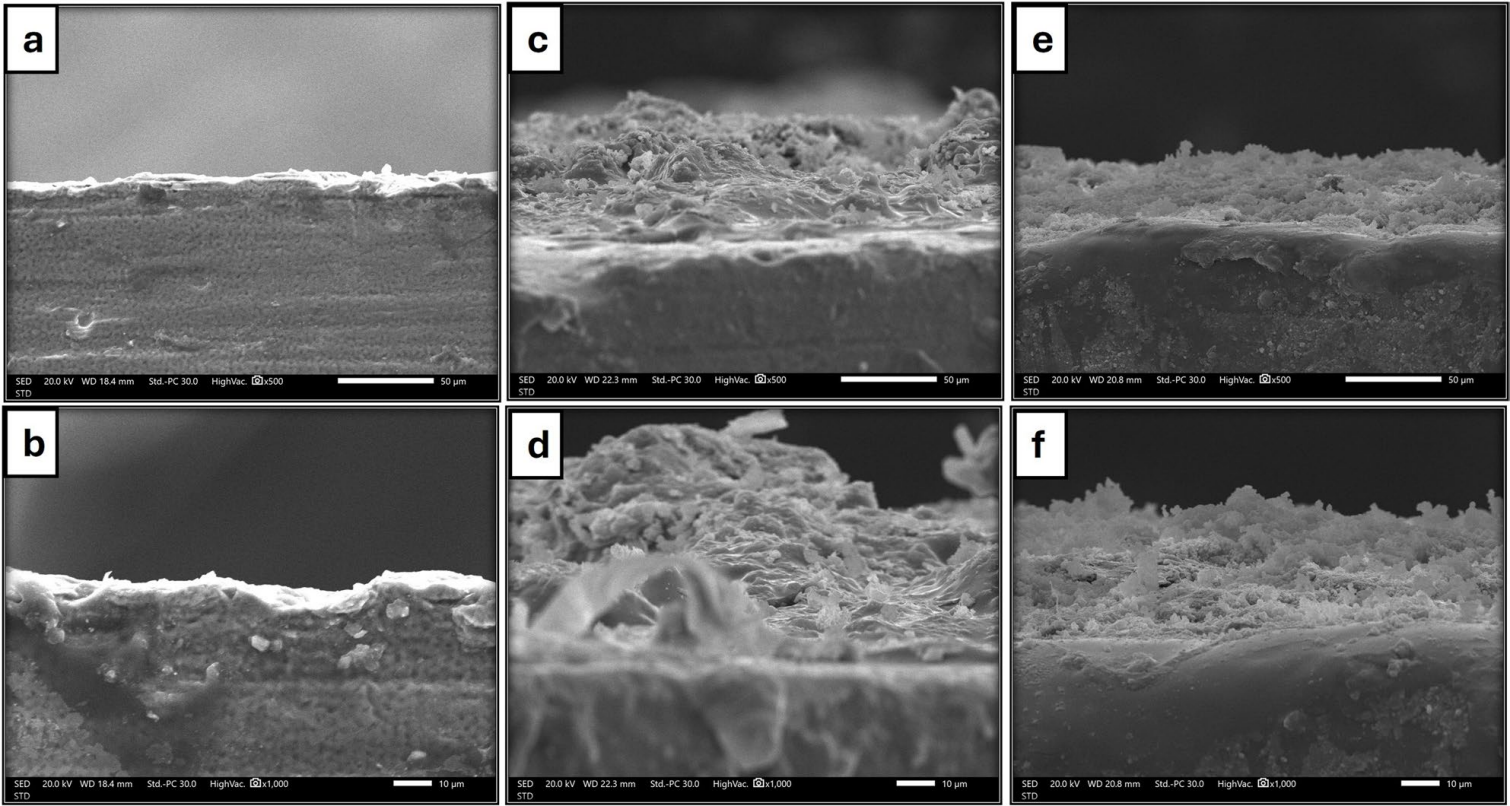
Cross-sectional scanning electron micrographs evaluating the coating thickness, homogeneity and substrate interface across different implant discs: **a**,** b** Uncoated titanium (Ti) disc demonstrating a clear boundary with no coating layer. **c**,** d** Carbon nanotube (CNT) coated titanium showing the thickness and adherence of the CNT layer to the underlying titanium substrate. **e**,** f** Carbon nanotube-1% hydroxyapatite (CNT-HA) nanocomposite coated titanium illustrating the structural integration, thickness, and uniform distribution of the nanocomposite layer on the titanium surface. Scale bars represent 50 μm at ×500 magnification for the top row (**a**, **c**, and **e**) and 10 μm at ×1000 magnification for the bottom row (**b**, **d**, and **f**)

The surface morphology of the coats on titanium was viewed using scanning electron microscope (JSM-IT200, JEOL, USA) and cross-sectional Scanning electron microscopic analysis was used to provide definitive data regarding coating thickness, homogeneity. Representative scanning electron micrographs of different surface coating morphology were presented in Figs. 1 and 2.

Besides, EDX spectra were acquired using the JSM-IT200 scanning electron microscope (JEOL, USA) equipped with an integrated EDX detector at the Electron Microscope Unit, Faculty of Science, Alexandria University. Analyses were performed at an accelerating voltage of 20 kV, a working distance of 10 mm, and a live acquisition time of approximately 30 s per spectrum. Elemental quantification was performed using the standardless ZAF correction method. The mean values were used to represent each group. Results are presented in Table 1.

**Table 1:**
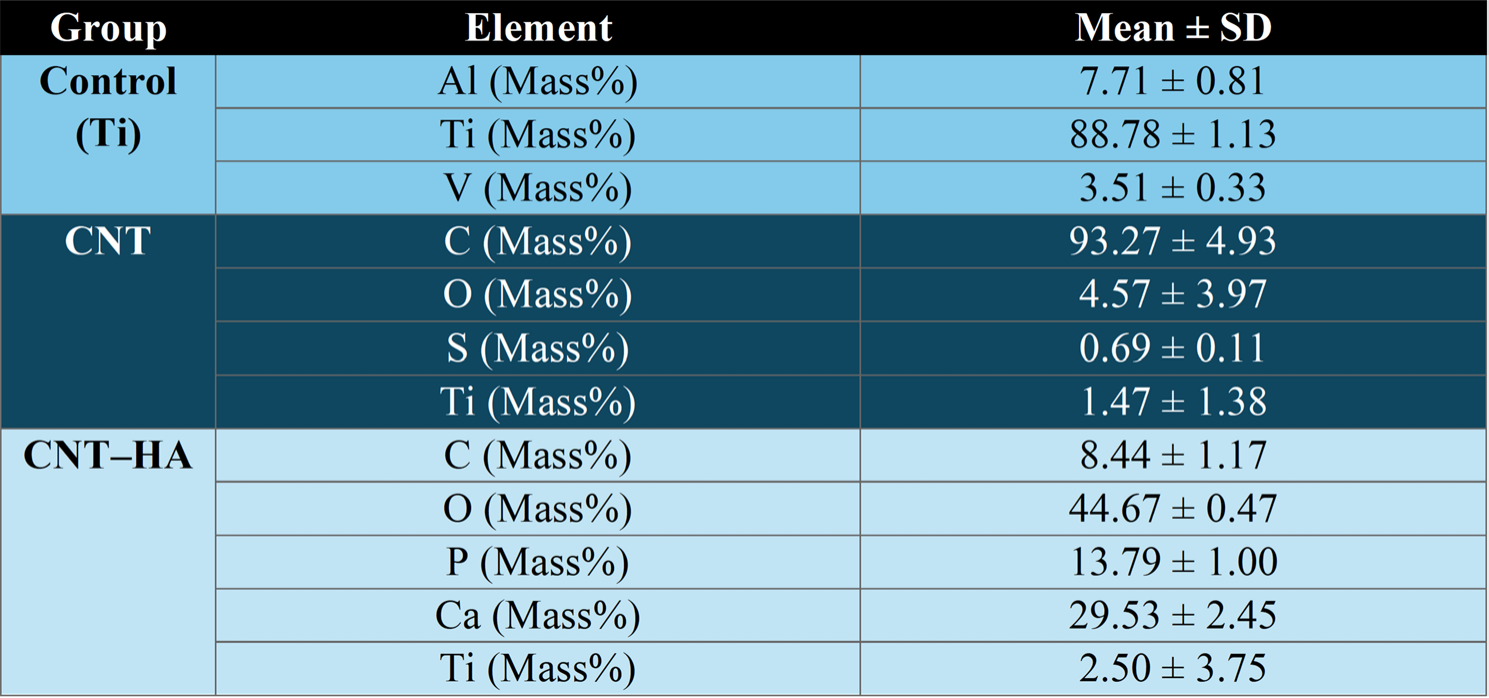
Energy Dispersive X-ray Spectroscopy (EDX) elemental composition (mass%) of uncoated titanium (Control/Ti), carbon nanotube-coated (CNT), and carbon nanotube–hydroxyapatite nanocomposite-coated (CNT–HA) implant surfaces. Mean ± SD column represents the mean and standard deviation across the specimens per group. The Control group shows elemental composition consistent with Ti-6Al-4 V alloy (Ti, Al, V). The CNT group is dominated by carbon with trace oxygen, sulfur, and titanium. The CNT–HA group demonstrates the characteristic elemental signature of hydroxyapatite (Ca, P, O) in addition to carbon, confirming successful nanocomposite coating

Also Surface characteristics were evaluated using confocal laser scanning microscopy (Leica TSC SPE II/DMi 8). Two-dimensional images of the implant surfaces were acquired, and surface profile analysis was performed using ImageJ software. Roughness-related parameters, including average roughness (Ra), root mean square roughness (Rq), maximum peak height (Rp), and maximum valley depth (Rv), were estimated from grayscale intensity profiles of the acquired images.

**Fig. 3:**
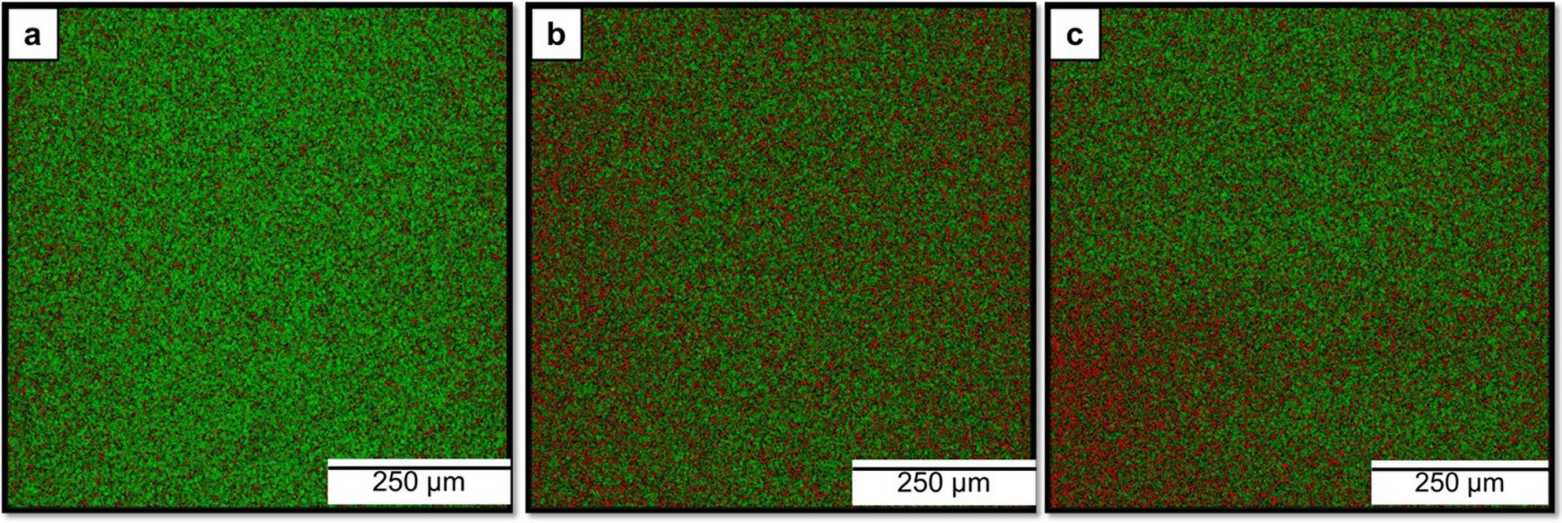
Confocal micrograph showing surface roughness of different coated implant discs: **a** uncoated titanium Ti, **b** Carbon nanotubes coated titanium CNT, and **c** Carbon nanotubes- 1% hydroxyapatite coated titanium CNT-HA

For each implant, measurements were obtained from multiple regions of interest, and the mean value was calculated to represent each sample. Representative confocal micrographs of the different groups were presented in Fig. 3.

### Surgical procedure

The selection, management, surgical protocols, and procedures for the animals were reviewed and approved by the Institutional Animal Care and Use Committee at Faculty of dentistry, Alexandria University, Egypt (ALEXU-IACUC-0832-12/2023) and are in adherence to the ARRIVE guidelines [22].

All surgical procedures were performed using sterile instruments and under strict aseptic conditions to minimize the risk of contamination during implantation.

**Fig. 4:**
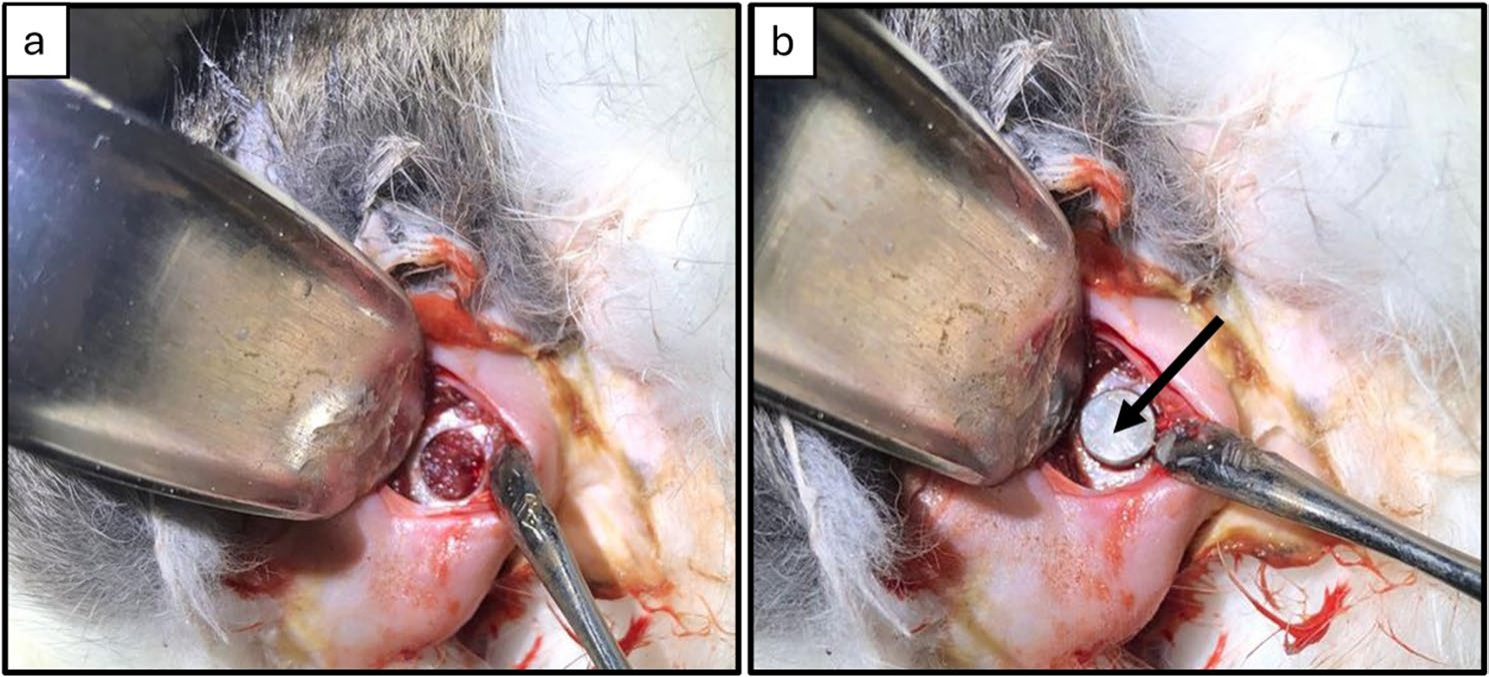
Surgical procedure (**a**) and site of implant disc (arrow) (**b**) in the angle of the mandible

Thirty adult male white rabbits (V-line), weighing 3–3.5 kg, were bought from the experimental Animal House, physiology department, Faculty of medicine, Alexandria University. The animals were anesthetized with intramuscular injections of Ketamine hydrochloride (Ketamine, Sigma tec, Egypt) (40 mg/kg), Xylazine hydrochloride (Xylaject, ADWIA Co., Egypt) (5 mg/kg) and were monitored. The areas near the angle of the mandible on both sides were shaved, disinfected with povidone iodine (Betadine, Mundipharma, Egypt) and 2 skin incisions were made to expose the angles of the mandible of each side and its anterior surface using scalpel number 15. The muscles were dissected and then periosteum was elevated to expose the bone surface of each angle of the mandible using periosteal elevators Fig. 4.

Next, two through and through bony defects were done with 6 mm trephine bur mounted on a low-speed handpiece, one on each side of the angle of the mandible and a sterile saline solution was used for cooling. The rabbits were randomly divided into three groups (10 rabbits per group) The rabbits were randomly divided into three groups (10 rabbits per group) using a computer-generated random number sequence (simple randomization) as follows: Group I (Control): uncoated titanium discs group (Ti), Group II: Carbon nanotubes coated titanium discs group (CNT), and Group III: Carbon nanotubes- 1% hydroxyapatite nanocomposite coated titanium group (CNT-HA). After that the titanium implant discs 3 mm in thickness & 6 mm in diameter[(Ti), (CNT), and (CNT-HA)] were implanted respectively in the bony defects with friction on each side of the angle of the mandible. After filling in the defects, the muscle and periosteum were repositioned, the deep layers were sutured with 3/0 polyglactin 910 suture (Vicryl, Ethicon, USA), and the skin was closed with interrupted 3/0 silk sutures (GMS, Ghatawry medical, Egypt). The surgical area was re-disinfected with povidone iodine (Betadine, Mundipharma, Egypt). Post-surgical care included the subcutaneous administration of 20 mL of normal saline and intramuscular Meloxicam (*Meloxicam*,* Nasr Pharmaceutical Company*,* Egypt*) (0.5 mg/kg/day) for 4 days, or until signs of pain resolved and each animal received intramuscular injection of Gentamicin sulfate antibiotic (Garamycin, Memphis Co. for Pharmaceutical & Chemical Industries, Egypt) (4 mg/kg/day) for 4 days following the surgery. Four weeks after implantation, all rabbits were euthanized, first they were deeply anesthetized using an intramuscular injection of Ketamine hydrochloride (Ketamine, Sigma tec, Egypt) (40 mg/kg) after that the animals were euthanized by an intravenous overdose of pentobarbital sodium “120 mg/Kg” (Nembutal, Akorn, Illinois, USA) administered through the marginal ear vein.

The harvested mandibular bone with implanted titanium discs were fixed in 10% neutral buffered formalin for 24 h, and processed to assess bone implant contact (BIC) and new bone area formation (NBA).

### Histo-pathological analysis

After fixation, the harvested samples were washed, dehydrated in a graded series of ethanol (40%, 70%, 95%, and 100%), cleared with xylene for 1 day and processed to be embedded in polymethyl methacrylate (PMMA) at room temperature. Following PMMA polymerization, the embedded samples were sliced along the buccolingual plane of the implant into sections measuring 200 μm in thickness using a precision cutting instrument (Microcut 150, Metkon Metallography, Turkey). These sections were stained with toluidine blue and Van Gieson-Stevenel’s blue stain to detect the bone-implant contact and the newly formed bone around the implants. The stained specimens were captured at ×40 and ×100 magnifications using a digital camera attached to an Optika B-290 series microscope (Ponteranica, Italy).

### Histo-morphometric analysis

Primary Endpoint: Osseointegration was primarily assessed by measuring the bone-to-implant contact ratio (BIC) as a percentage of the implant interface in direct contact with bone. This parameter was designated as the primary endpoint and was used as the basis for sample size estimation.

Secondary Endpoint: Additionally, the newly formed bone area (NBA) was measured as a pre-specified secondary outcome to evaluate the percentage of new bone area to the entire tissue area within a 1 mm ring region around the implant.”

For each implant, three representative undecalcified sections were obtained and analyzed using ImageJ software. Measurements of BIC and NBA were performed for each section, and the mean value was calculated to represent each implant. Statistical analysis was conducted using the implant (animal) as the experimental unit (*n* = 10 per group).

### Statistical analysis

Data are presented as mean ± standard deviation (SD). For histomorphometric analysis, the measurements obtained from the three sections per implant were averaged to yield a single representative value for each implant. These values were used for statistical comparison between groups.

Statistical differences among the three groups were analyzed using one-way analysis of variance (ANOVA), followed by Tukey’s post hoc multiple comparisons test. Statistical analysis was performed using PSPP software (version 1.6.2). A p-value < 0.05 was considered statistically significant.

## Results

### Surface topography and roughness analysis

Representative scanning electron micrographs of different surface coating morphology of implant discs were presented in Fig. 1 and 2.

EDX analysis confirmed the elemental composition of each implant surface group (Table 1). The Control (Ti) group demonstrated a composition consistent with Ti-6Al-4 V alloy, comprising predominantly titanium (88.78 ± 1.13 mass%), with aluminum (7.71 ± 0.81 mass%) and vanadium (3.51 ± 0.33 mass%), and no carbon signal detectable above background. The CNT-coated group was dominated by carbon (93.27 ± 4.93 mass%), confirming successful deposition of the carbon nanotube coating. Trace amounts of oxygen (4.57 ± 3.97 mass%) and sulfur (0.69 ± 0.11 mass%) were detected, likely reflecting residual functional groups introduced during the acid functionalization step, and a minor titanium signal (1.47 ± 1.38 mass%) indicated partial substrate signal contribution at the 20 kV beam energy used. The CNT–HA group exhibited a distinctly different profile, characterized by prominent oxygen (44.67 ± 0.47 mass%), calcium (29.53 ± 2.45 mass%), and phosphorus (13.79 ± 1.00 mass%) signals alongside carbon (8.44 ± 1.17 mass%), consistent with the presence of hydroxyapatite within the nanocomposite coating. The calculated Ca/P molar ratio derived from atomic percentages across the specimens ranged from approximately 1.64 to 1.67, in close agreement with the stoichiometric value of 1.67 for hydroxyapatite, thereby confirming the phase purity of the synthesized HA component.

While the Surface roughness and characteristics were evaluated using confocal laser scanning microscopy, and the surface roughness parameters (Ra, Rq, Rp, and Rv) are summarized in Table 2. All values are expressed in micrometers (µm) as mean ± standard deviation (SD).

**Table 2:**
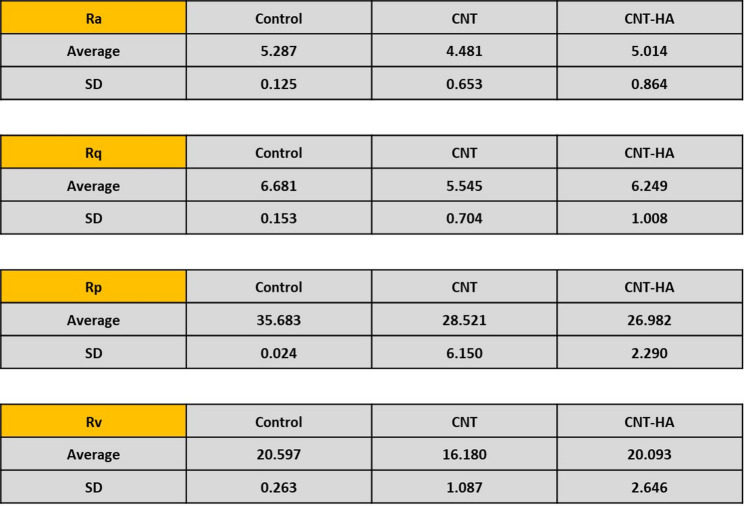
Surface roughness parameters of uncoated titanium (Ti), carbon nanotube-coated (CNT), and carbon nanotube–hydroxyapatite-coated (CNT–HA) implant groups measured using confocal laser scanning microscopy. Parameters include average roughness (Ra), root mean square roughness (Rq), maximum peak height (Rp), and maximum valley depth (Rv). Data are presented as mean ± standard deviation (SD) in micrometers (µm)

Quantitative analysis revealed variations in roughness parameters among the groups. The uncoated titanium group exhibited the highest average roughness (Ra = 5.29 ± 0.13 μm), followed by the CNT–HA-coated group (Ra = 5.01 ± 0.86 μm), while the CNT-coated group demonstrated a lower mean roughness value (Ra = 4.48 ± 0.65 μm). A similar trend was observed for Rq values.

Regarding surface profile extremes, the uncoated titanium group showed the highest maximum peak height (Rp = 35.68 μm), whereas the CNT–HA group exhibited a reduced Rp value (26.98 μm). In addition, the CNT-coated group demonstrated the lowest maximum valley depth (Rv = 16.18 μm) compared to the control group (Rv = 20.60 μm), indicating a relatively more uniform surface profile.

Overall, the application of CNT and CNT–HA coatings resulted in modifications of the surface profile characteristics of titanium implants, with variations observed in both average roughness and peak–valley features. However, these changes did not follow a consistent trend of increasing roughness across all parameters.

### Histological and histomorphometric findings

**Fig. 5:**
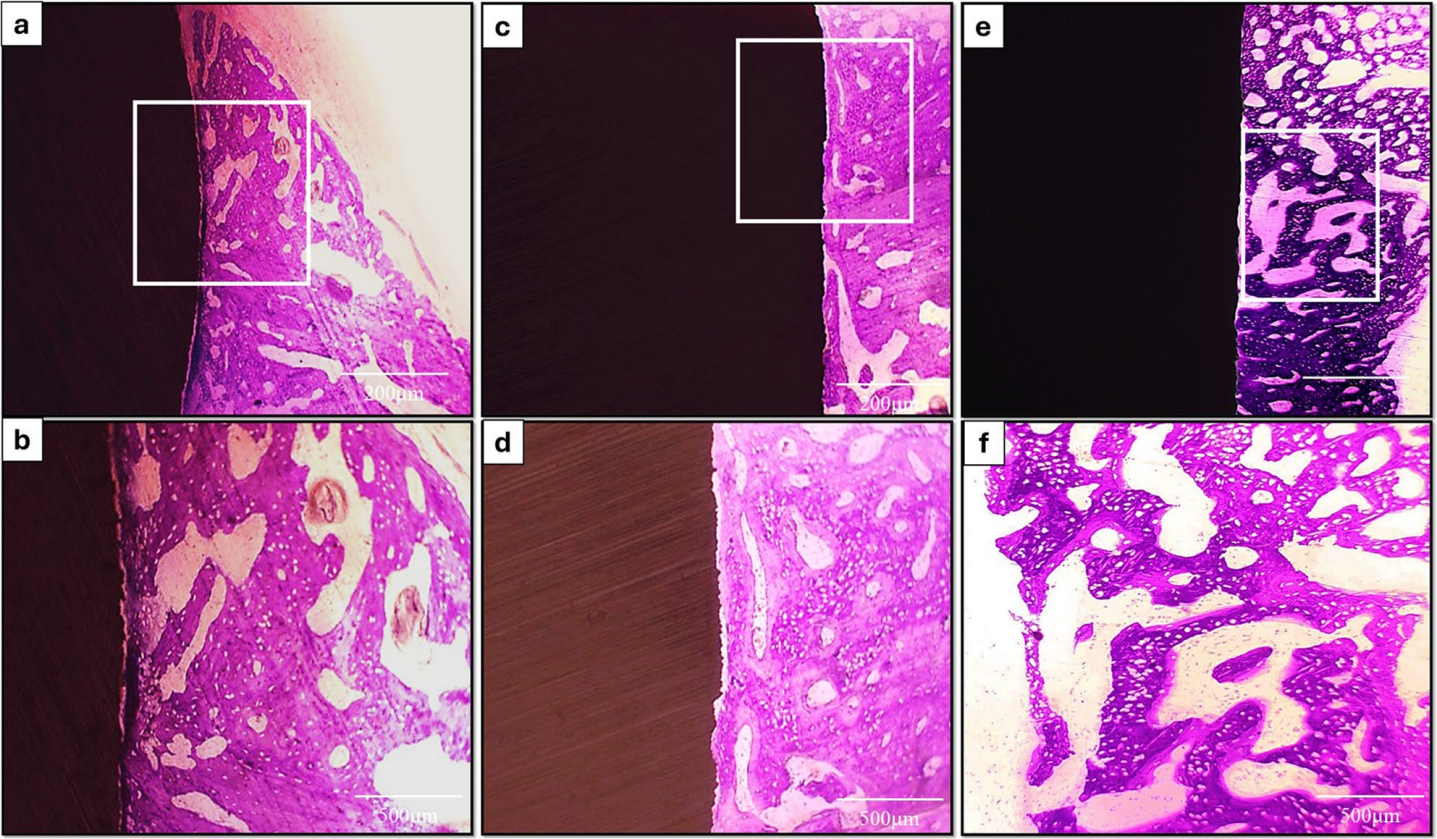
Representative Toluidine blue -stained photomicrographs of titanium implant discs 4 weeks after implantation. **a**, **b** uncoated titanium Ti, **c**, **d** Carbon nanotubes coated titanium CNT, and **e**, **f** Carbon nanotubes- 1% hydroxyapatite coated titanium CNT-HA. Scale bar = 500 μm is ×40 (**a**, **c**, and **e**), while scale bar = 200 μm is ×100 (**b**, **d**, and **f**)

**Fig. 6:**
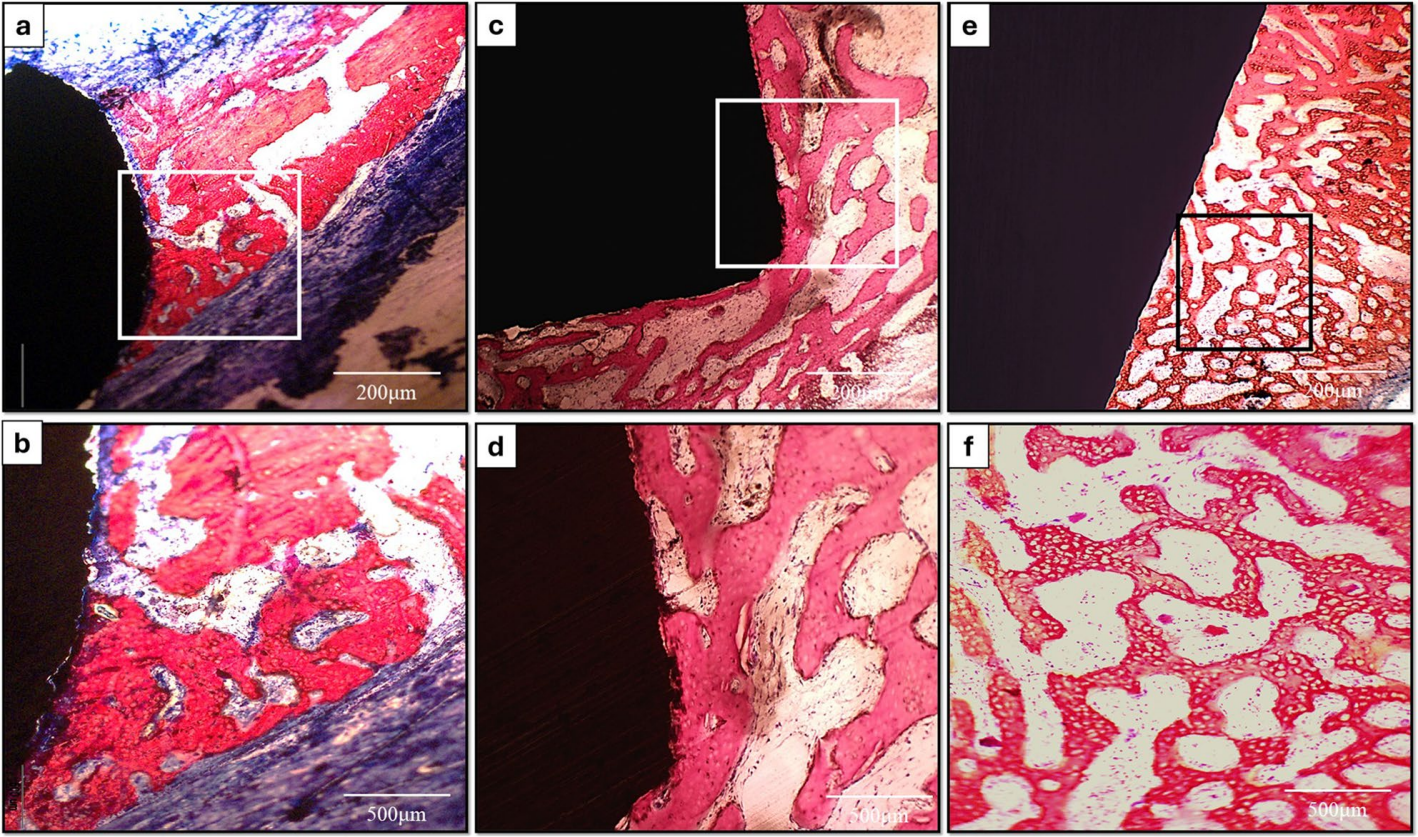
Representative Van Gieson-Stevenel’s blue -stained photomicrographs of titanium implant discs 4 weeks after implantation. **a**, **b** uncoated titanium Ti, **c**, **d** Carbon nanotubes coated titanium CNT, and **e**, **f** Carbon nanotubes- 1% hydroxyapatite coated titanium CNT-HA. Scale bar = 500 μm is ×40 (**a**, **c**, and **e**), while scale bar = 200 μm is ×100 (**b**, **d**, and **f**)

**Fig. 7:**
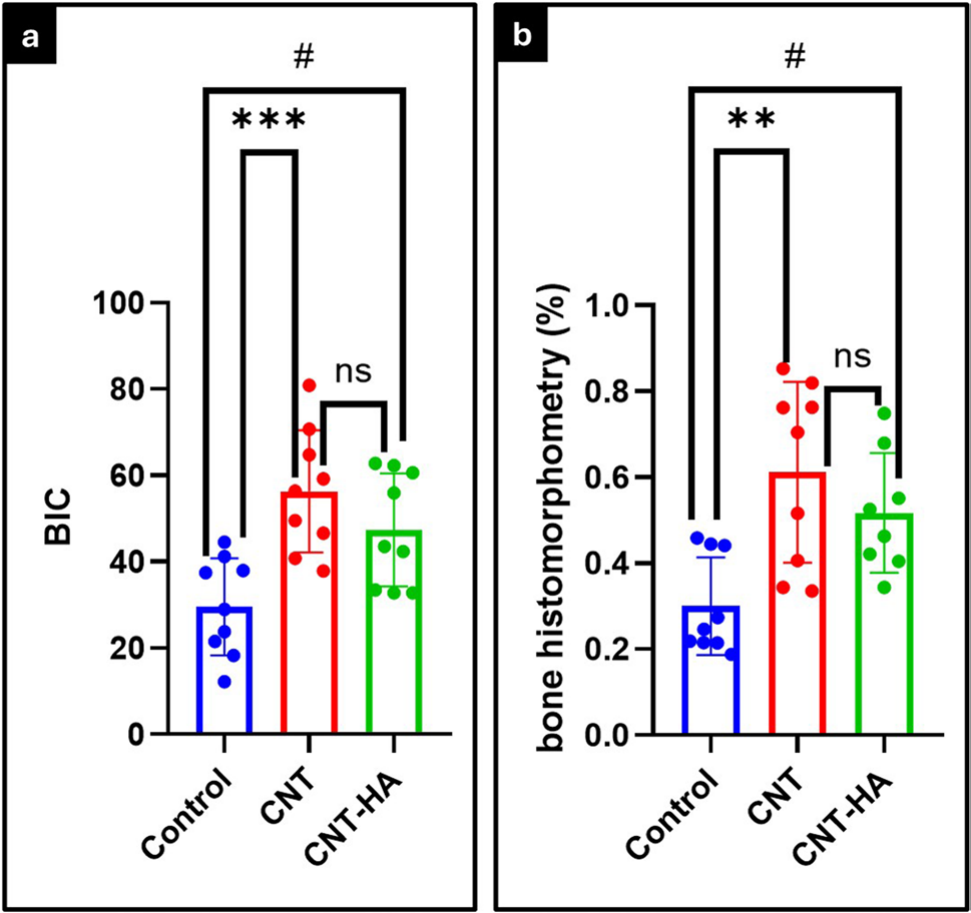
The graphs of BIC (**a**), and NBA (**b**); show higher BIC and NBA for the CNT implant compared with Ti, CNTs-HA implants, *P* < 0.05

Representative histological sections of the peri-implant regions at 4 weeks were illustrated in Figs. 5 and 6, while quantitative histomorphometric measurements of bone–implant contact (BIC) and new bone area (NBA) were presented in Fig. 7.

Histological examination revealed clear differences in peri-implant tissue responses among the experimental groups. The uncoated titanium (Ti) group frequently exhibited incomplete osseointegration, characterized by the presence of fibrous connective tissue at the bone–implant interface and limited new bone formation adjacent to the implant surface. In contrast, both the carbon nanotube (CNT) and carbon nanotube–hydroxyapatite nanocomposite (CNT–HA) coated implants showed more favorable osseointegration, with newly formed bone observed in direct contact with the implant surfaces at multiple sites. These coated groups displayed well-organized bone trabeculae, an absence of inflammatory cell infiltration, and a greater amount of lamellar bone containing numerous osteocytes compared to the uncoated Ti group.

Histomorphometric analysis confirmed these qualitative findings. The CNT group exhibited the highest mean BIC value (56.30% ± 14.19%), which was significantly greater than that of the Ti control group (29.54% ± 11.28%; *P* = 0.0005). The CNT–HA group also demonstrated a significantly higher BIC (47.41% ± 13.09%) compared with uncoated titanium (*P* = 0.0191). Although the CNT group had a higher mean BIC than the CNT–HA group, this difference was not statistically significant (*P* = 0.3265), indicating that both coatings performed similarly in terms of bone–implant contact. Similarly, analysis of new bone formation revealed significantly greater NBA values in both coated groups compared with the Control Ti group. The CNT group showed the highest mean NBA (0.6116 ± 0.2106), followed by the CNT–HA group (0.5171 ± 0.1395), while the control Ti group demonstrated the lowest value (0.3002 ± 0.1137). Statistically significant differences were observed between the control Ti group and both CNT (*P* = 0.0012) and CNT–HA groups (*P* = 0.0279). No statistically significant difference in NBA was detected between the CNT and CNT–HA groups (*P* = 0.4592).

Overall, both CNT-based coatings significantly enhanced early peri-implant bone formation and osseointegration compared with uncoated titanium implants, with CNT showing the highest numerical values for both BIC and NBA, although these differences were not statistically significant compared to the CNT–HA group.

## Discussion

This experimental study evaluated the effect of carbon nanotube (CNT) and carbon nanotube–hydroxyapatite (CNT–HA) surface coatings on the osseointegration of titanium implant discs in a rabbit mandibular model. The results demonstrated that both CNT and CNT–HA coatings significantly improved early osseointegration, as evidenced by increased bone–implant contact (BIC) and new bone area (NBA), compared with uncoated titanium, demonstrating the bioactive potential of carbon nanomaterials in enhancing osseointegration. This finding is consistent with previous reports showing that carbon nanomaterial coatings on titanium implants can promote early bone formation and improve bone–implant integration. Kang et al. (2021) reported that carbon nanomaterial coatings enhanced cellular attachment and proliferation on titanium surfaces, thereby improving osseointegration in both dental and orthopedic applications [23]. Similarly, Salou et al. (2015) observed that nanostructured titanium surfaces increased bone formation and implant stability in a rabbit model, highlighting the role of surface nano topography in facilitating osteoconduction [24].

However, the addition of hydroxyapatite to CNT coatings did not result in a statistically significant increase in BIC or NBA compared to CNT alone, which aligns with previous observations that while HA is osteoconductive, its benefits may plateau when combined with other highly bioactive nanomaterials [25]. Qiang et al. demonstrated that HA composite coatings improve bone formation on titanium alloys; however, the synergistic effect with other nanomaterials may depend on factors such as coating thickness, uniformity, and early-stage cellular interactions [26]. This may explain why no further enhancement in osseointegration was observed with CNT–HA compared to CNT coatings in our study.

Uncoated titanium implants exhibited incomplete osseointegration with areas of fibrous tissue interposition, reflecting the bioinert nature of titanium surfaces despite their favorable biocompatibility [27]. Surface modification strategies have therefore been widely explored to enhance the biological interaction between titanium implants and surrounding bone by modifying surface chemistry and topography [28]. In the present study, CNT-based coatings resulted in direct bone–implant contact without inflammatory reactions, indicating a more favorable healing environment.

The CNT group demonstrated the highest mean BIC and NBA values, suggesting superior early bone response. While the precise mechanisms underlying this enhanced performance were not directly investigated in the present study, it is hypothesized on the basis of published evidence that the nanoscale architecture and high surface area of carbon nanotubes may facilitate protein adsorption and osteoblast adhesion, as reported in prior in vitro and in vivo investigations. The presence of oxygen-containing functional groups on the CNT surface, as partially evidenced by the oxygen signal detected on EDX in the present study, may also contribute to improved biological interactions; however, direct biochemical evidence such as protein adsorption assays or cell attachment studies was not obtained here, and these proposed mechanisms should therefore be regarded as working hypotheses rather than conclusions. The nanoscale cues provided by CNTs may contribute to accelerated early bone formation, as suggested by the histological sections [29]. CNTs have been shown to provide extracellular matrix-like topographical cues that accelerate early bone formation and promote stable osseointegration [30]. These findings suggest that enhanced early bone response may be beneficial in situations where early implant loading is desired. Although CNT–HA implants showed significantly better osseointegration than uncoated titanium, their performance was not statistically superior to CNT/Ti implants. Hydroxyapatite is a well-established osteoconductive material that promotes bone apposition due to its chemical similarity to bone mineral [31]. However, the absence of a significant difference between CNT and CNT–HA groups suggests that the addition of hydroxyapatite at the tested concentration (1%) did not provide an additional early-stage benefit. This may be explained by the dominant influence of CNT-induced nanotopography on early cellular responses, potentially masking the osteoconductive contribution of hydroxyapatite during the initial healing period. Similar findings have been reported in previous studies, where CNT-based coatings alone elicited strong early osteogenic responses [20].

Regarding surface characteristics, confocal microscopy analysis revealed variations in surface roughness parameters (Ra, Rq, Rp, and Rv) among the experimental groups. Notably, the CNT-coated group demonstrated a lower average roughness compared to uncoated titanium, while the CNT–HA group exhibited values comparable to the control. Despite these findings, both coated groups showed enhanced osseointegration, indicating that the biological improvements observed in this study cannot be attributed solely to surface roughness. Rather, these results suggest that nanoscale surface characteristics and surface chemistry play a more critical role in modulating the bone response [23]. Carbon nanotubes are known to enhance protein adsorption and promote osteoblast adhesion and proliferation due to their high surface energy and nanoscale architecture [6], while hydroxyapatite contributes to osteoconductivity by mimicking the mineral phase of bone and facilitating bone bonding. Therefore, the improved osseointegration observed may be primarily driven by bioactive and nanotopographical effects rather than changes in conventional roughness parameters. It should also be noted that roughness parameters were estimated from two-dimensional confocal images, which may limit the accuracy of absolute roughness quantification.

EDX analysis provided important evidence for the successful deposition and chemical integrity of the coatings. The elemental profile of the Control group confirmed the expected Ti-6Al-4 V alloy composition, validating the substrate material used. In the CNT group, the carbon-dominant spectrum with trace oxygen and sulfur signals reflects the outcome of the acid functionalization step, during which carboxyl and hydroxyl groups are introduced onto the CNT surface. This surface functionalization is considered critical for improving the dispersibility, biocompatibility, and bonding potential of CNTs to titanium substrates, as well as for facilitating protein adsorption and subsequent cell attachment. The presence of residual titanium signal in the CNT group is consistent with signal penetration through the coating at 20 kV beam energy, and does not indicate incomplete coating; rather, it reflects the relatively thin nature of the electrophoretically deposited layer relative to the interaction volume of the electron beam. In the CNT–HA group, the characteristic Ca and P signals, combined with a Ca/P molar ratio of approximately 1.67, confirm the stoichiometric hydroxyapatite phase within the nanocomposite, validating the wet-chemical precipitation synthesis protocol. The reduced carbon signal in this group compared to the CNT group is attributable to the incorporation of HA particles, which partially displace carbon from the surface layer detected by EDX. Taken together, the EDX findings confirm that the coating deposition process was chemically consistent across specimens, and that the elemental compositions are aligned with the intended coating materials, supporting the validity of the biological outcomes observed.

From a preliminary standpoint, these findings suggest that CNT-only surface modification shows potential for enhancing early osseointegration. However, further long-term studies and biomechanical evaluations are required to confirm the functional stability of these coatings under loading conditions. This has practical implications, as simpler coating systems may reduce manufacturing complexity and improve coating stability, while avoiding potential drawbacks associated with hydroxyapatite coatings such as brittleness and delamination [32]. Therefore, CNT-based coatings may represent a promising and efficient approach for improving implant integration. The rabbit mandibular model employed in this study is well-suited for evaluating early osseointegration, given its high bone turnover rate and similarities to human cortical bone remodeling [14]. The use of undecalcified histological sections combined with quantitative histomorphometric analysis enabled precise assessment of peri-implant bone response. Furthermore, the absence of inflammatory reactions in this study corroborates previous findings regarding the biocompatibility of functionalized CNTs as implant surface coatings [33].

## Limitations

While the present study provides valuable insight into the biological performance of CNT-based coatings, several limitations should be acknowledged. First, comprehensive physicochemical characterization of the coatings was not performed for the specific experimental batch. Although surface profile parameters (Ra, Rq, Rp, and Rv) were estimated using confocal microscopy, these measurements were derived from two-dimensional images and do not fully represent three-dimensional surface topography or chemical composition. While EDX analysis confirmed the surface elemental composition of all groups (Table 1). FTIR spectroscopy for functional group identification, XRD for phase analysis and crystallinity assessment, and Raman spectroscopy for carbon structure confirmation were not conducted. These techniques would be necessary to fully validate the physicochemical integrity, homogeneity, and adhesion properties of the CNT and CNT–HA coatings. This represents a recognized limitation of the present study, and future investigations should incorporate such analyses to provide more complete coating validation.

In addition, only a single concentration of hydroxyapatite was evaluated, which may not fully capture its potential dose-dependent effects. The use of a rabbit model, while suitable for assessing early osseointegration, does not fully replicate long-term clinical conditions or functional loading environments. Furthermore, UVC irradiation was employed as a surface decontamination step rather than a validated sterilization method, which should be addressed in future studies using standardized sterilization protocols or microbiological validation.

Despite these limitations, the study design allowed for controlled comparative evaluation among groups, and the consistent improvement in osseointegration parameters supports the potential of CNT-based coatings as bioactive implant surface modifications.

## Conclusion

In conclusion, within the limitations of this study, carbon nanotube (CNT)-based coatings significantly enhanced early osseointegration of titanium implants compared with uncoated surfaces. CNT coatings alone achieved comparable outcomes to CNT–HA coatings, indicating that carbon nanotubes are sufficient to promote early peri-implant bone formation, while the addition of hydroxyapatite at the tested concentration did not provide a statistically significant additional benefit.

Importantly, the enhanced biological response occurred independently of measurable changes in surface roughness. While EDX analysis confirmed the surface elemental composition consistent with the intended coating materials, the specific nanoscale and biochemical mechanisms underlying the improved osseointegration were not directly characterized in this study and therefore cannot be conclusively attributed to any particular surface property. These findings nonetheless highlight the potential of CNT-based surface modifications for improving early implant integration, and further studies incorporating comprehensive physicochemical characterization, optimized coating compositions, and long-term functional evaluation are required to confirm their clinical applicability and elucidate the underlying mechanisms.

## Data Availability

The datasets used and/or analyzed during the current study are available from the corresponding author on reasonable request.

## References

[1] Chen Q, Thouas GA. Metallic implant biomaterials. Mater Sci Engineering: R: Rep. 2015;87:1–57.

[2] Saini M, Singh Y, Arora P, Arora V, Jain K. Implant biomaterials: A comprehensive review. World J Clin Cases. 2015;3(1):52–7. 25610850 10.12998/wjcc.v3.i1.52 PMC4295219

[3] Wennerberg A, Albrektsson T. Effects of titanium surface topography on bone integration: a systematic review. Clin Oral Implants Res. 2009;20:172–84. 19663964 10.1111/j.1600-0501.2009.01775.x

[4] Dorozhkin SV. Calcium orthophosphate coatings on magnesium and its biodegradable alloys. Acta Biomater. 2014;10(7):2919–34. 24607420 10.1016/j.actbio.2014.02.026

[5] Albrektsson T, Wennerberg A. Oral implant surfaces: Part 1--review focusing on topographic and chemical properties of different surfaces and in vivo responses to them. Int J Prosthodont. 2004;17(5). 15543910

[6] Eatemadi A, Daraee H, Karimkhanloo H, Kouhi M, Zarghami N, Akbarzadeh A, Abasi M, Hanifehpour Y, Joo SW. Carbon nanotubes: properties, synthesis, purification, and medical applications. Nanoscale Res Lett. 2014;9(1):393. 25170330 10.1186/1556-276X-9-393 PMC4141964

[7] Kou W, Akasaka T, Watari F, Sjogren G. An in vitro evaluation of the biological effects of carbon nanotube-coated dental zirconia. ISRN Dent. 2013;2013:296727. 24027638 10.1155/2013/296727 PMC3762083

[8] Newman P, Minett A, Ellis-Behnke R, Zreiqat H. Carbon nanotubes: their potential and pitfalls for bone tissue regeneration and engineering. Nanomedicine. 2013;9(8):1139–58. 23770067 10.1016/j.nano.2013.06.001

[9] Mocan T, Matea CT, Pop T, Mosteanu O, Buzoianu AD, Suciu S, Puia C, Zdrehus C, Iancu C, Mocan L. Carbon nanotubes as anti-bacterial agents. Cell Mol Life Sci. 2017;74(19):3467–79. 28536787 10.1007/s00018-017-2532-y PMC11107489

[10] Saliev T. The Advances in Biomedical Applications of Carbon Nanotubes. C. 2019;5(2):29.

[11] Saifuddin N, Raziah AZ, Junizah AR. Carbon Nanotubes: A Review on Structure and Their Interaction with Proteins. J Chem. 2013;2013:676815.

[12] Dorozhkin SV. Bioceramics of calcium orthophosphates. Biomaterials. 2010;31(7):1465–85. 19969343 10.1016/j.biomaterials.2009.11.050

[13] LeGeros RZ. Properties of osteoconductive biomaterials: calcium phosphates. Clin Orthop Relat Research^®^. 2002;395:81–98. 10.1097/00003086-200202000-00009 11937868

[14] Suo L, Jiang N, Wang Y, Wang P, Chen J, Pei X, Wang J, Wan Q. The enhancement of osseointegration using a graphene oxide/chitosan/hydroxyapatite composite coating on titanium fabricated by electrophoretic deposition. J Biomedical Mater Res Part B: Appl Biomaterials. 2019;107(3):635–45. 10.1002/jbm.b.34156 29802685

[15] Charan J, Biswas T. How to calculate sample size for different study designs in medical research? Indian J Psychol Med. 2013;35(2):121–6. 24049221 10.4103/0253-7176.116232 PMC3775042

[16] Pannucci CJ, Wilkins EG. Identifying and avoiding bias in research. Plast Reconstr Surg. 2010;126(2):619–25. 20679844 10.1097/PRS.0b013e3181de24bc PMC2917255

[17] Faul F, Erdfelder E, Lang A-G, Buchner A. G* Power 3: A flexible statistical power analysis program for the social, behavioral, and biomedical sciences. Behav Res Methods. 2007;39(2):175–91. 17695343 10.3758/bf03193146

[18] Park JE, Jang YS, Bae TS, Lee MH. Biocompatibility characteristics of titanium coated with multi walled carbon nanotubes—hydroxyapatite nanocomposites. Materials. 2019 Jan 10;12(2):224. 10.3390/ma12020224 PMC6356870 30634682

[19] Mondal S, Dey A, Pal U. Low temperature wet-chemical synthesis of spherical hydroxyapatite nanoparticles and their in situ cytotoxicity study. Adv Nano Res. 2016;4(4):295–307.

[20] Ibrahim Y, Kamoun E, Abdel Moaty M, Mohy El Din M. Evaluation of carbon nanotubes-hydroxyapatite nanocomposites as bioactive implant coats radiated by near infrared laser. Eur J Oral Sci. 2022;130(4):e12873. 35673772 10.1111/eos.12873

[21] Riley DJ, Bavastrello V, Covani U, Barone A, Nicolini C. An in-vitro study of the sterilization of titanium dental implants using low intensity UV-radiation. Dent Mater. 2005;21(8):756–60. 15878616 10.1016/j.dental.2005.01.010

[22] Percie du Sert N, Hurst V, Ahluwalia A, Alam S, Avey MT, Baker M, Browne WJ, Clark A, Cuthill IC, Dirnagl U. The ARRIVE guidelines 2.0: Updated guidelines for reporting animal research. J Cereb Blood Flow Metab. 2020;40(9):1769–77. 32663096 10.1177/0271678X20943823 PMC7430098

[23] Kang MS, Lee JH, Hong SW, Lee JH, Han D-W. Nanocomposites for enhanced osseointegration of dental and orthopedic implants revisited: Surface functionalization by carbon nanomaterial coatings. J Compos Sci. 2021;5(1):23.

[24] Salou L, Hoornaert A, Louarn G, Layrolle P. Enhanced osseointegration of titanium implants with nanostructured surfaces: An experimental study in rabbits. Acta Biomater. 2015;11:494–502. 25449926 10.1016/j.actbio.2014.10.017

[25] Marasli C, Katifelis H, Gazouli M, Lagopati N. Nano-Based Approaches in Surface Modifications of Dental Implants: A Literature Review. Molecules. 2024;29(13):3061. 10.3390/molecules29133061 PMC11243276 38999015

[26] Qiang W, Jin Z, Lijun Z, Shimin L, Yanqin L. Investigation and application of HA composite coating on the Ti alloy. Hydroxyapatite Coatings Biomed Applications. 2013:361.

[27] Saini M, Singh Y, Arora P, Arora V, Jain K. Implant biomaterials: A comprehensive review. World J Clin Cases: WJCC. 2015;3(1):52. 25610850 10.12998/wjcc.v3.i1.52 PMC4295219

[28] Marenzi G, Impero F, Scherillo F, Sammartino JC, Squillace A, Spagnuolo G. Effect of different surface treatments on titanium dental implant micro-morphology. Materials. 2019;12(5):733. 30836588 10.3390/ma12050733 PMC6427554

[29] Parwez K, Budihal SV. Carbon nanotubes reinforced hydroxyapatite composite for biomedical application. J Bionanosci. 2014;8(1):61–5.

[30] Newman P, Minett A, Ellis-Behnke R, Zreiqat H. Carbon nanotubes: their potential and pitfalls for bone tissue regeneration and engineering. Nanomed Nanotechnol Biol Med. 2013;9(8):1139–58. 10.1016/j.nano.2013.06.001 23770067

[31] Ahmed F, Rashid H, Farookhi S, Verma V, Khalifa M, Sheikh Z, Dh D. Surface modifications of endosseous dental implants by incorporation of roughness and hydroxyapatite coatings. JPDA. 2015;24(04):162.

[32] Shin US, Yoon I-K, Lee G-S, Jang W-C, Knowles JC, Kim H-W. Carbon nanotubes in nanocomposites and hybrids with hydroxyapatite for bone replacements. J tissue Eng. 2011;2011:674287. 21776341 10.4061/2011/674287 PMC3138058

[33] Kou W, Akasaka T, Watari F, Sjögren G. An in vitro evaluation of the biological effects of carbon nanotube-coated dental zirconia. Int Sch Res Notices. 2013;2013(1):296727. 10.1155/2013/296727 PMC3762083 24027638

